# Cultivable fungal diversity in two karstic caves in Italy: under-investigated habitats as source of putative novel taxa

**DOI:** 10.1038/s41598-024-54548-1

**Published:** 2024-02-20

**Authors:** A. Poli, A. Zanellati, E. Piano, F. Biagioli, C. Coleine, G. Nicolosi, L. Selbmann, M. Isaia, V. Prigione, G. C. Varese

**Affiliations:** 1 Department of Life Sciences and Systems Biology, Mycotheca Universitatis Taurinensis, University of Torino, Viale Mattioli 25, 10100 Torino, Italy; 2 Department of Life Sciences and Systems Biology, University of Torino, Via Accademia Albertina 13, 10123 Torino, Italy; 3 Department of Ecological and Biological Sciences, University of Tuscia, Largo Dell’Università, 01100 Viterbo, Italy

**Keywords:** Leotiomycetes, Mycobiota, Phylogeny, Show-cave, Ecosystem ecology, Molecular ecology, Fungal ecology, Microbial communities, Environmental microbiology

## Abstract

Microbial diversity of caves is largely understudied and its possible applications are still unknown. Autochthonous fungi, in particular, may have the potential to biomineralize metals and may be used as promising agents for bioremediation of polluted sites; thus, unearthing the fungal diversity in hypogean ecosystems is nowadays of utmost importance. To start addressing this knowledge gap, the cultivable mycobiota of two neighbouring caves—one natural and one exploited for touristic purposes—were characterised and compared by studying fungi isolated from sediments collected at increasing distances from the entrance. Overall, 250 fungal isolates ascribable to 69 taxa (mainly Ascomycota) were found, a high percentage of which was reported in caves for the first time. The sediments of the touristic cave displayed a richer and more diversified community in comparison with the natural one, possibly due to visitors carrying propagules or organic material. Considering that these environments are still poorly explored, chances to detect new fungal lineages are not negligible.

## Introduction

Caves are confined oligotrophic subterranean environments that, being characterised by constant low temperature, high humidity and darkness, can be considered extreme^1–3^. Indeed, in natural caves, three different areas are defined by light penetration and intensity: the entrance zone, the twilight zone and the dark zone. Contrary to the first two zones where light penetrates directly or indirectly, the darkest part of the caves display more extreme conditions due to lack of photosynthesis and consequently of nutrients^4^. All these factors contribute to shaping a unique ecosystem where only highly adapted organisms can settle^5,6^.

The usage of caves for touristic purposes, together with climate change effects, are the major threats to subterranean diversity^7–10^. To date, the so-called show caves count 1440 sites in 148 countries (www.showcaves.com), and the massive and constant presence of outsiders causes fluctuation of temperature and humidity^11,12^, and impact on geochemical properties^13^. Indeed, through their skin, shoes, clothes, and litter left behind, visitors can spread propagules into the cave, thus altering the natural microbial community^14,15^. In addition, the cave ecosystem is severely affected by the artificial lights installed through the touristic path: the so-called “lampenflora” consists of biofilms of phototrophic organisms that develop on illuminated surfaces^16–20^.

Recently, the effect of human disturbance on the microbial communities was demonstrated by analysing the sediments of four touristic and one natural caves in Italy through next generation sequencing^20^. The authors observed that, while tourism pressure directly and indirectly affected bacteria, fungi and archaea responded only to changes in sediment composition induced by human presence^21^.

Fungi, in particular, are keystone components of the subterranean microbiota, considering that more than 1600 species in 640 genera have been reported from caves and mines worldwide^22–25^. Functioning as parasites, decomposers, or serving as food for other organisms, cave fungi occur in various substrates (e.g. sediments, rocks, mineral deposits, guano etc.) mainly as spores, carried in by water, air currents, or animals^25,26^. These entrance routes are the reason why the greatest diversity is generally recovered from the entrance and twilight zones^27–29^. Understanding the fungal biodiversity in hypogean ecosystems and its role in ecological and geological processes is getting more and more attention, also considering that autochthonous fungi may be biotechnologically exploited as a source of novel active compounds^30^.

Despite the increasing interest, most of the previous studies were focused on cave fauna and bacteria, while fungal diversity has often been neglected^31^.

To address this knowledge gap, the present work aimed to: (i) unveil and compare the cultivable fungal diversity in a touristic cave and in a natural one; (ii) uncover possible autochthonous fungal species and potential novel lineages; (iii) determine the effect of tourists on allochthonous fungi colonisation.

## Results

All sampling sites were colonized by fungi. The colonization rate ranged from 7899 CFU g dw^−1^ (Sector 3, 37 °C) and 9,018,718 CFU g dw^−1^ (Sector 2, 24 °C) (Table 1). The biodiversity indices, namely Pielou’s evenness (J′), Simpson (1-Lambda), and the Shannon–Wiener diversity (H′) were higher in CC (Table 1).

**Table 1:** Average fungal load (CFU g dw^-1^ ± SEM) in different sites for each incubation temperature. Biodiversity indeces within sampling sites: Shannon–Weaver index (H’), Gini-Simpson index (1-Lambda) and Pielou’s evenness (J’).

	S1	S2	S3	S4	Bossea Tot	CC
10 °C	3.9 × 10^5^ ± 1.9 × 10^5^	1.8 × 10^5^ ± 7.0 × 10^4^	2.5 × 10^5^ ± 9.5 × 10^4^	1.7 × 10^5^ ± 1.2 × 10^4^		8.3 × 10^4^ ± 3.1 × 10^4^
25 °C	6.5 × 10^6^ ± 5.2 × 10^6^	9.0 × 10^6^ ± 4.9 × 10^6^	8.9 × 10^5^ ± 6.0 × 10^5^	3.6 × 10^6^ ± 2.1 × 10^6^		1.4 × 10^5^ ± 5.8 × 10^4^
37 °C	2.2 × 10^5^ ± 2.0 × 10^5^	1.4 × 10^5^ ± 8.7 × 10^4^	7.9 × 10^3^ ± 5.4 × 10^3^	0		0
Diversity indeces						
Total taxa	19	28	31	20	60	9
H′	0.3826	0.8107	1.759	0.5463	1.126	2.281
1-l′	0.1321	0.3515	0.621	0.1828	0.4508	0.8432
J′	0.1299	0.2433	0.5121	0.1824	0.275	0.8423

Overall, 250 isolates ascribable to 69 taxa were retrieved from the two caves (Table 2). Out of these, 63 were identified at species level, while four and two remained at genus and class level, respectively. In total, 197 sequences (94 nrITS, 11 nrLSU, 3 nrSSU, 61 alpha-actin, 25 beta-tubuline and 3 RPB2) were newly generated and 71 were deposited in Genbank. The dominant phylum was Ascomycota (min. 80% in CC—max. 100% in S4) followed by Basidiomycota (min. 0% in S4—max. 20% in CC). Chytridiomycota, Mucororomycota and Rozellomycota were not detected.

**Table 2:** Fungal taxa isolated: * from one sampling site exclusively; ^*■*^ at 10 °C; ° at 25 °C; ^+^ at 37° C. Taxa with no sign were isolated at both 10 °C and 25 °C. FR = First Report in caves worldwide.

Taxon	S1	S2	S3	S4	CC	FR from caves	Ref
**Ascomycota**							
*Alternaria alternata*	✕	–	✕	✕	–		^25^
Anopodium ampullaceum*	✕	–	–	–	–	FR	This study
*Arthroderma terrestre*^■^*	✕	–	–	–	–	FR	This study
*Arthroderma uncinatum*^■^*	–	✕	–	–	–		^26^
*Aspergillus fumigatus*^+^*	–	✕	–	–	–		^25^
*Aspergillus terreus*^+^	✕	✕	✕	–	–		^25^
Aspergillus tubingensis°*	–	✕	–	–	–		^25^
*Aureobasidium pullulans*	✕	–	✕	✕	✕		^32^
*Biscogniauxia nummularia*	–	–	–	–	✕	FR	This study
Botrytis cinerea*	–	–	✕	–	–		^32^
Cadophora dextrinospora*	–	–	✕	–	–	FR	This study
*Cadophora* sp.*	–	–	✕	–	–		^25,33^
*Cephalotrichum domesticum*^*■*^	–	–	✕	✕	–	FR	This study
*Cephalotrichum longicollum*^■^*	–	✕	–	–	–	FR	This study
*Cladosporium allicinum*^*■*^	–	✕	–	✕	✕		Nováková et al. (2018)
*Cladosporium cladosporioides*	✕	✕	✕	✕	✕		^25^
*Cladosporium halotolerans*	✕	✕	✕	✕	–		^25^
*Cladosporium perangustum*	✕	✕	–	✕	–		^25^
*Cladosporium pseudocladosporioides*	✕	✕	✕	✕	✕		^25^
*Cladosporium* sp.*	–	–	–	–	✕		^25,26,33^
Cordyceps farinosa*	–	–	–	–	✕		^23^
Curvularia americana°*	✕	–	–	–	–	FR	This study
*Epicoccum nigrum*	✕	✕	✕	✕	–		^25^
Furcasterigmium furcatum*	–	–	✕	–	–		^25,26^
*Fusarium* sp*.*^■^*	–	–	–	✕	–		^26,31,33^
Gibellulopsis nigrescens*	–	–	✕	–	–		^25,26^
Heterosporicola beijingensis*	–	–	–	✕	–	FR	This study
Lasionectria hilhorstii*	–	–	✕	–	–	FR	This study
*Lecanicillium coprophilum*^■^*	–	✕	–	–	–		^25^
*Phacidiales* sp.^■^*	–	–	–	✕	–	FR	This study
*Leotiales* sp.^■^*	–	–	✕	–	–	FR	This study
Leptosphaeria ogilviensis*	–	✕	–	–	–	FR	This study
*Mammaria echinobotryoides*	✕	–	✕	–	–		^26^
*Microdochium nivale*^■^*	–	–	✕	–	–		^26^
*Myrmecridium sambuci*^■^*	–	–	–	–	✕	FR	This study
*Neobulgaria* sp*.*^■^*	–	–	–	✕	–	FR	This study
Oidiodendron tenuissimum*	–	–	✕	–	–		^26^
*Paracremonium variiforme*^*■*^	–	✕	–	✕	–		^25^
Paraleptosphaeria macrospora*	✕	–	–	–	–	FR	This study
*Penicillium antarcticum*	✕	✕	✕	✕	–		^9^
Penicillium brevicompactum°*	–	–	✕	–	–		^25^
Penicillium chrysogenum°*	–	✕	–	–	–		^25^
Penicillium concentricum°*	–	–	–	–	✕		^25^
*Penicillium expansum*	–	✕	✕	–	–		^25^
*Penicillium glabrum*	✕	–	✕	✕	–		^25^
*Penicillium glandicola*^■^*	–	–	–	–	✕		^25^
*Penicillium griseofulvum°*	–	✕	✕	✕	–		^32^
*Penicillium rubens*	✕	–	✕	✕	–		^25^
Penicillium steckii°*	–	–	✕	–	–		^32^
Periconia pseudobyssoides°*	–	✕	–	–	–	FR	This study
Phaeosphaeria glyceriae-plicatae°*	–	–	–	✕	–	FR	This study
*Pichia manshurica*^+^*	–	✕	–	–	–	FR	This study
*Pseudogymnoascus pannorum*^*■*^	✕	✕	✕	–	✕		^25^
Sarocladium subulatum°*	–	–	–	–	✕		^34^
*Sporothrix inflata*^■^*	✕	–	–	–	–		^25^
*Stephanonectria keithii*^■^*	–	–	✕	–	–		^25^
*Tetracladium globosum*^*■*^	–	–	✕	✕	–	FR	This study
Tolypocladium cylindrosporum*	–	✕	–	–	–		^25^
Trichocladium asperum*	–	–	✕	–	–		^31^
*Trichosporiella cerebriformis*	–	✕	✕	–	–		^26^
*Volutella ciliata*^■^*	–	✕	–	–	–		^25^
**Basidiomycota**							
*Apiotrichum dulcitum*^■^*	–	✕	–	–	–		^26,33^
Candolleomyces candolleanus °*	–	✕	–	–	–		^26,31^
Coprinellus micaceus°*	–	–	✕	–	–		^26^
Daedaleopsis confragosa*	–	✕	–	–	–	FR	This study
Fomitopsis palustris*	–	✕	–	–	–	FR	This study
Moesziomyces bullatus°*	✕	–	–	–	–	FR	This study
Peniophora crystallina°*	–	–	–	–	✕	FR	This study
*Rhodosporidiobolus odoratus*^■^*	–	–	–	–	✕	FR	This study

The isolated taxa were affiliated to 10 classes, 22 orders, 35 families and 49 genera.

### Taxonomic distribution among sectors

**Figure 1:**
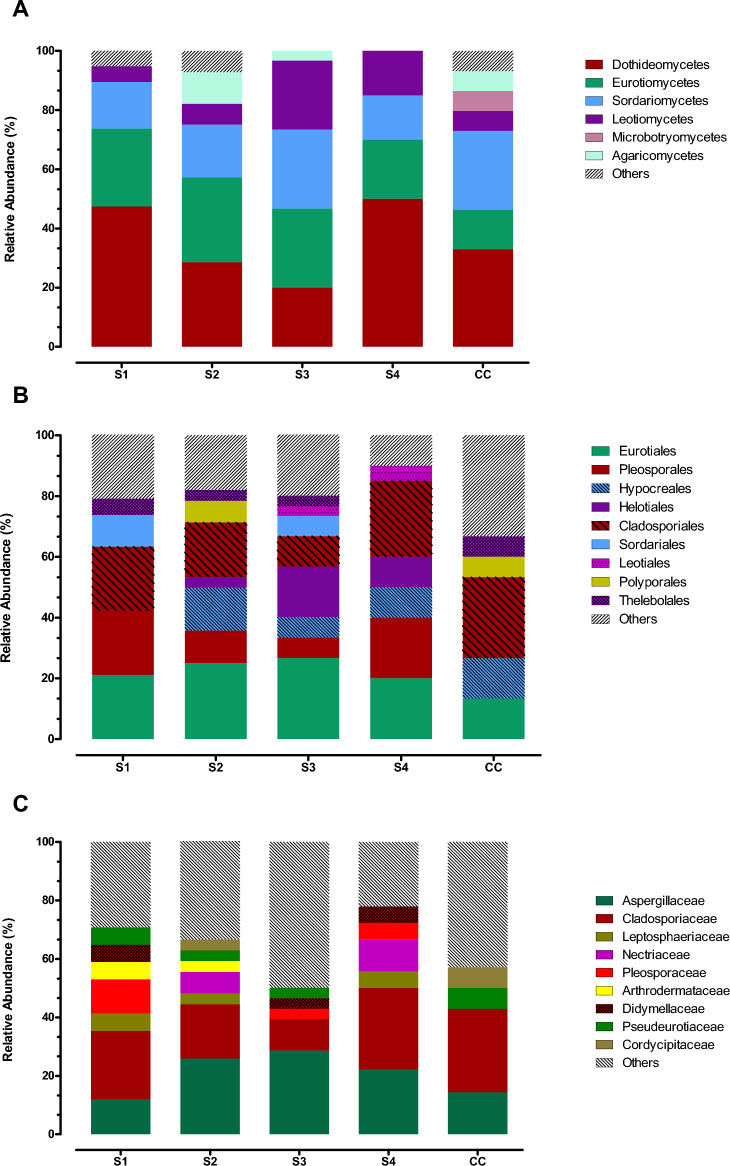
Relative abundance of fungi retrieved in the 4 sectors of Bossea (S1, S2, S3 and S4) and Costacalda (CC) caves, (**A**) Distribution in classes; (**B**) Distribution in orders; (**C**) Distribution in families.

The best represented classes were Dothideomycetes (Min 20% S3—Max 50% S4), Eurotiomycetes (Min 20% S4—Max 28.6% S2), and Sordariomycetes (Min 15.8% S1—Max 26.7% S3 and CC), followed by Leotiomycetes (Min 2.7% S2—Max 23.3% S3). Agaricomycetes were detected in S2 (10.7%), S3 (3.3%) and CC (5.7%). Microbotryomycetes accounted for 6.7% and were detected only in CC, while Saccharomycetes, Tremellomycetes and Ustilaginomycetes were only marginally represented in S1 and S2 (“Others”, Fig. 1A). Among the more abundant orders, Cladosporiales (Min 10%—Max. 27%) and Eurotiales (Min 13.3%—Max. 27%) occurred in all sampling sites, while the order Pleosporales was not detected in CC (Fig. 1B). Agaricales, Dothideales, Entylomatales, Glomerellales, Microascales, Myrmecridiales, Onygenales, Ophiostomatales, Saccharomycetales, Sporidiobolales, Trichosporonales, Ustilaginales, Xylariales accounted together for up to 33% (CC) and were grouped together (“Others”, Fig. 1B).

Aspergillaceae, likewise Cladosporiaceae, were found across all samples and range from 11.8% (S1) to 28.6% (S3), and from 10.7% (S3) to 28.6% (CC), respectively. Arthrodermataceae, Didymellaceae, Leptosphaeriacea, Nectriaceae, and Pleosporaceae were present in lower percentages in at least two sectors of Bossea cave and were not observed in CC. Taken together, Ploettnerulaceae, Plectosphaerellaceae, Bionectriaceae, Microascaceae, Saccotheciaceae Lasiosphaeriaceae, Psathyrellaceae, Periconiaceae, Phaeosphaeriaceae, Sclerotiniaceae, Helotiaceae, Pseudeurotiaceae, Saccharomycetaceae, Ophiocordycipitaceae, Ophiostomataceae, Chaetomiaceae, Microdochiaceae, Myxotrichaceae, Fomitopsidaceae, Polyporaceae, Trichosporonaceae, Ustilaginaceae, Sarocladiaceae, Myrmecridiaceae, Graphostromataceae, Meruliaceae, Sporidiobolaceae accounted for up to 50% (S3) (“Others”, Fig. 1C).

*Cladosporium* and *Penicillium* were the most common genera and were found in all sites (Table 2) with a relative abundance that ranged from 10% (S3) to 26.7% (CC) and from 13.3% (CC) to 23.3% (S3), respectively.

**Figure 2:**
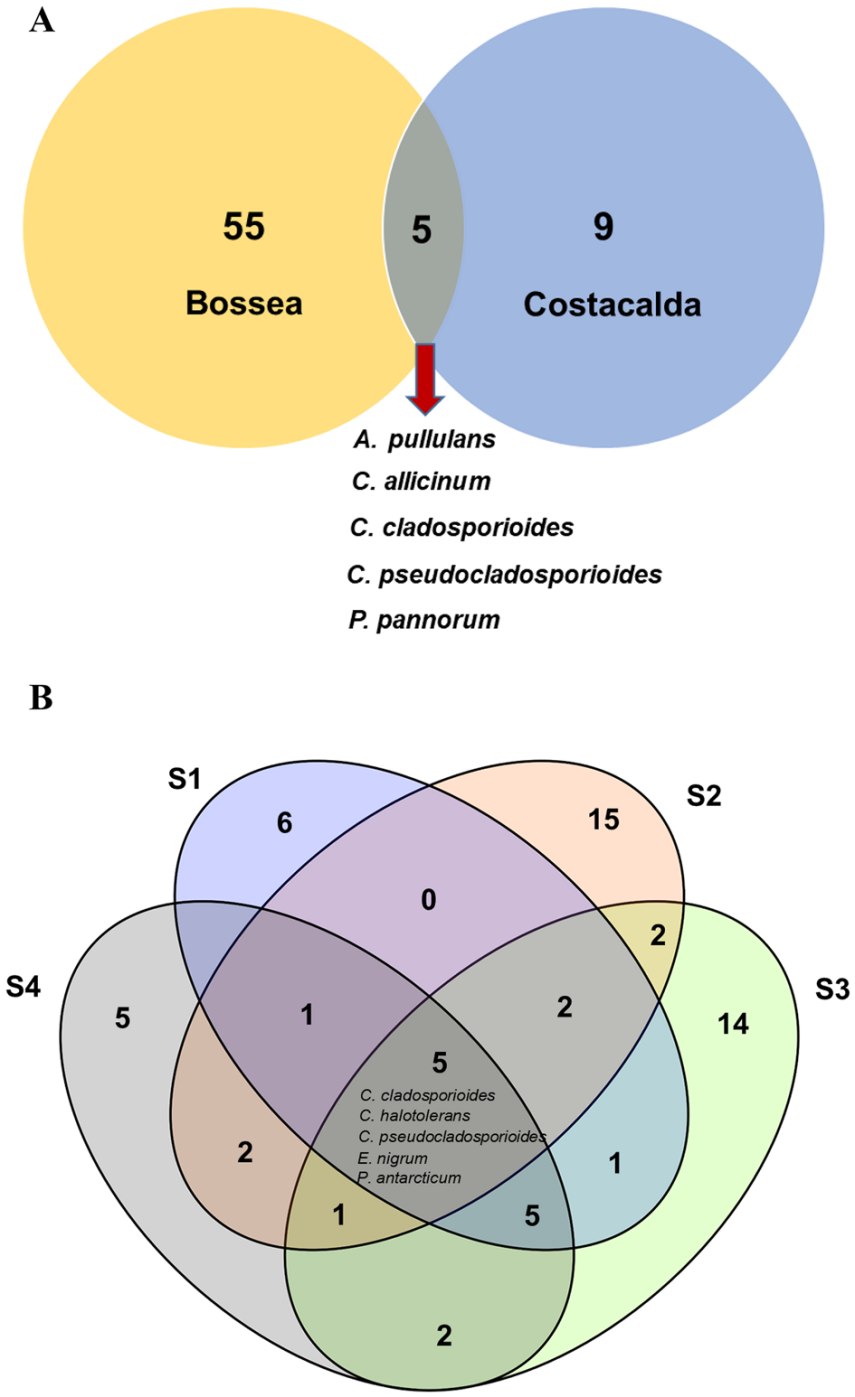
Venn diagram showing the total number of taxa and shared taxa between the caves of Bossea and Costacalda (**A**) and among the four sectors of Bossea (**B**).

Only two species, namely *Cladosporium cladosporioides* and *Cladosporium pseudocladosporioides*, were common to all sites (S1, S2, S3, S4 and CC; Table 2). A few species were shared between at least two sampling sites, while 9 (*Biscogniauxia nummularia*, *Cladosporium* sp., *Cordyceps farinosa*, *Myrmecridium sambuci*, *Penicillium concentricum*, *Penicillium glandicola*, *Rhodosporidiobolus odoratus*, *Sarocladium subulatum* and *Scopuloides rimosa*) and 55 taxa were exclusively isolated from CC and Bossea caves, respectively. The two caves shared *Aurobasidium pullulans*, *Cladosporium allicinum*, *C. cladosporioides*, *C. pseudocladosporioides* and *Pseudogymnoascus pannorum* (Fig. 2A). Considering the cave of Bossea alone, five taxa were common to the four sectors (*C. cladosporioides*, *Cladosporium halotolerans*, *C. pseudocladosporioides*, *Epicoccum nigrum* and *Penicillium antarcticum*), while 6, 15, 14 and 12 were exclusively isolated from S1, S2, S3 and S4, respectively (Fig. 2B).

### Fungal community

**Figure 3:**
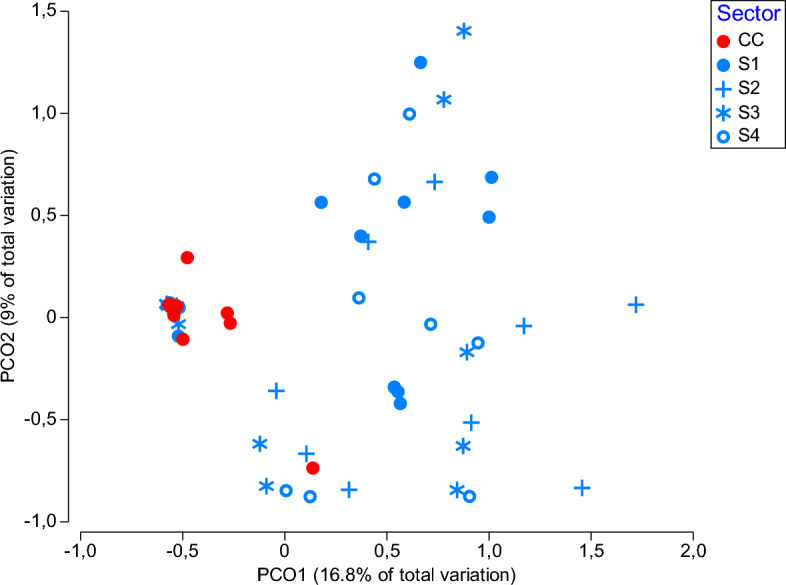
Analysis of Principal coordinates (PCO) illustrating the diversity of fungal communities among sampling sites.

In terms of fungal species diversity, the four sectors of Bossea showcave were not different among each other (PERMANOVA; *p* > 0.05; Fig. 3), while each of them was significantly distinct from Costacalda cave (PERMANOVA; *p* < 0.05; Fig. 3). The most frequently retrieved species in the cave of Bossea were *C. halotolerans* (44%), followed by *C. pseudocladosporioides* (16%) and *P. antarcticum* (5%), while *Cordyceps farinosa* (44%) and *P. pannorum* (35%) were better represented in Costacalda (SIMPER analysis).

The fungal community of Bossea showcave included three strains that remained identified as *Neobulgaria* sp. (MUT 6739; isolated from S4) and *Leotiomycetes* sp. (MUT 6736 and MUT 6737 isolated from S3 and S4, respectively) and that are representative of possible new lineages, as detailed below.

### Phylogenetic inference

MUT 6739 was initially identified as a member of Gelatinodiscaceae (Helotiales, Leotiomycetes) based on BLASTn analysis of nrITS, nrSSU nrLSU and RPB2 (Table S1). Due to the scarcity in GenBank of RPB2 and nrSSU sequences for this family, only nrITS and nrLSU were considered for further analyses. Analogously, MUT 6736 and MUT 6737 were grouped into the orders Leotiales and Phacidiales (Leotiomycetes) following inspection of BLASTn hits of nrITS, nrLSU, nrSSU and RPB2 (Table S1). As in the previous case, RPB2 sequences were scant. Consequently, phylogenetic inference was focused on the three ribosomal markers.

Preliminary analyses carried out individually with nrITS and nrLSU for Gelatinodiscaceae, and nrITS, nrSSU, and nrLSU for Leotiales/Phacidiales, revealed no incongruence in the topology of the single-loci trees. The combined datasets were built on the basis of the BLASTn results and the most recent phylogenetic studies on Leotiomycetes (Quijada et al. 2018, Ekanayaka et al. 2019, Johnston et al. 2019, Quijada et al. 2022).

**Figure 4:**
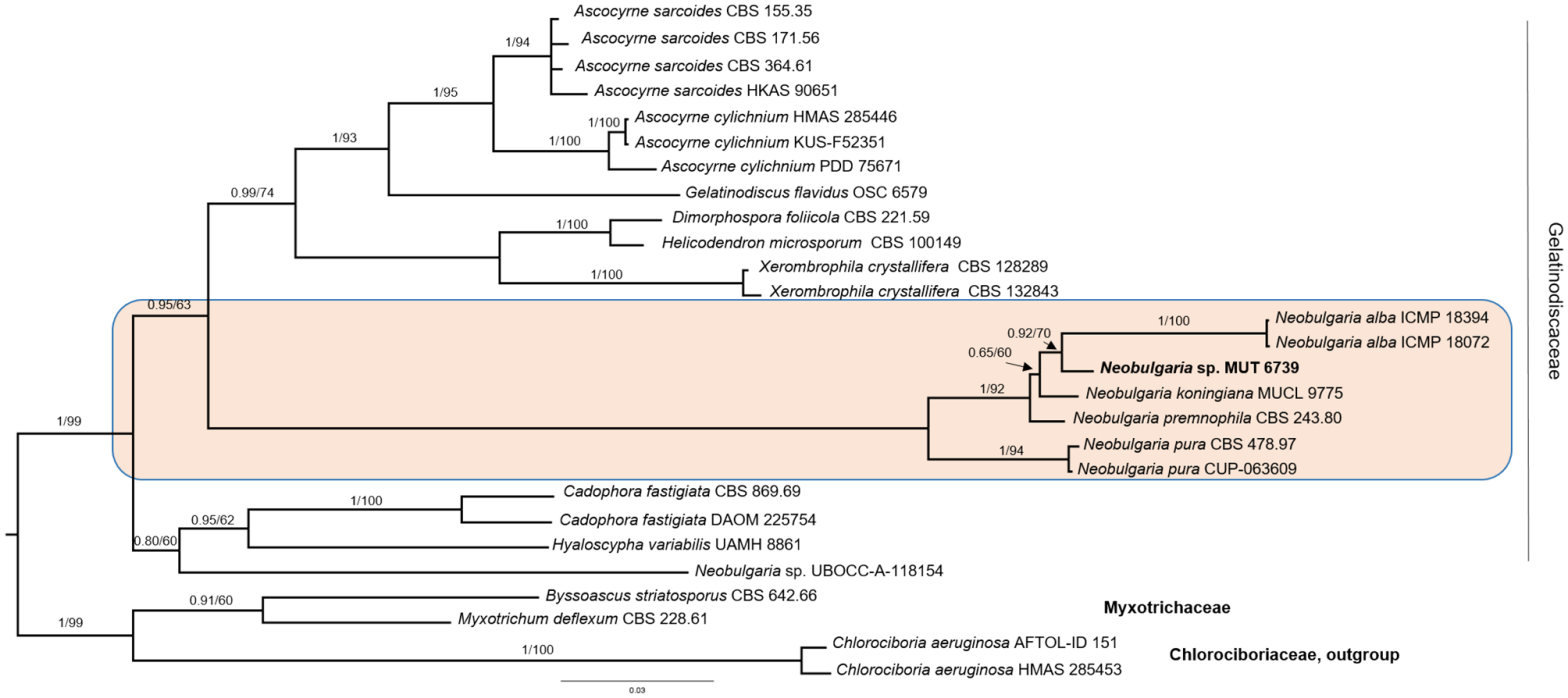
Bayesian phylogram of Gelatinodiscaceae based on a combined nrITS, and nrLSU dataset. The tree is rooted to *Chlorociboria aeruginosa*. Branch numbers indicate BYPP/BS values; Bar = expected changes per site (0.03).

The dataset for Gelatinodiscaceae consisted of 27 taxa, including MUT 6739, that represented 11 genera and 16 species (Table 3). The alignment was 1,248 characters long; 841 sites were conserved, 64 were parsimony uninformative and 343 parsimony informative (TL = 879, CI = 0.560886, RI = 0.793880, HI = 0.439114). MUT 6739 grouped into the Neobulgaria clade (BYPP = 1, BS = 100), however, although closely related, MUT 6739 was distant from any known species and represented a putative novel lineage (Fig. 4).

**Table 3:** Dataset used for phylogenetic analysis. Genbank sequences include newly generated nrITS, nrLSU and nrSSU amplicons relative to the putative novel species.

Taxon	Strain	ITS	SSU	LSU
Helotiales				
Gelatinodiscaceae				
* Ascocoryne cylichnium*	PDD7567	AY789395	–	–
	KUS-F52351	JN033406	–	JN086709
	HMAS 90651	MK584973	KR094137	OQ534476
* Ascotremella faginea*	JAC14763	MK432811	–	–
* Ascocoryne sarcoides*	CBS364.61	MH858085	–	MH869655
	CBS171.56	MH857563	–	MH869105
	CBS155.35	MH855613	–	MH867123
	HKAS 90651	MK584973	MK585054	MK591999
* Ascocoryne solitaria*	CBS:738.84	HM152545	DQ002904	–
* Byssoascus striatosporus*	CBS 642.66	MH858902	NG_070873	MH870573
* Cadophora fastigiata*	CBS:869.69	MH859469	–	MH871247
	DAOM 225754	JN942894	JN939030	JN938877
* Chlorociboria aeruginosa*	HMAS 285453	OQ534206	–	OQ534492
	AFTOL-ID 151	DQ491501	AY544713	AY544669
* Dimorphospora foliicola*	CBS 221.59	MH857844	–	MH869385
* Gelatinodiscus flavidus*	OSC 6579	EU652349	–	EU652381
* Helicodendron microsporum*	CBS:100149	MH862690	KR078445	KR078441
* Hyaloscypha variabilis*	UAMH 8861	NR_121313		NG_073616.1
* Myxotrichum deflexum*	CBS 228.61	LN833542	NG_065476	MH872267
* Neobulgaria alba*	ICMP 18394	NR_137054	HM116781	–
	ICMP18072	HM116745	HM116761	–
* Neobulgaria koningiana*	MUCL 9775	NR_165900	MK185672	MK185694
* Neobulgaria premnophila*	CBS 243.80	MH861260	U45445	MH873029.1
* Neobulgaria lilacina*	M258	–	EU940066	EU940141
* Neobulgaria pura*	CBS 478.97	JN033385	–	JN086688
	CUP-063609	DQ257366	DQ257364	DQ257365
* Neobulgaria* sp*.*	UBOCC-A-118154	–	–	MT226563
* Neocudoniella radicella*	UAMH 5794	NR_121301	AY524843	–
* Xerombrophila crystallifera*	CBS128289	MH864847	–	MH876294
	CBS 132843	JX481974	–	MH878488
**Leotiales**				
Leotiales i.s				
* Alatospora acuminata*	CBS 104.88	MH862121	–	MH873811
	CCM-F 02383	AY204587	–	KC834018
* Alatospora pulchella*	CCM F-502	KC834039	–	KC834019
* Flagellospora curvula*	CB_M13	KC834045	MK226450	KC834024
* Collophora paarla*	CBS 120878	GQ154575	GQ154632	GQ154611
* Pallidophorina paarla*	CBS 120877 Type	NR_119749	GQ154634	MK314610
Leotiaceae				
* Leotia lubrica*	KKM 427	KF836621	–	KF836631
* Microglossum rufum*	AFTOL-ID 1292	DQ257360	DQ471033	DQ470981
* Microglossum olivaceum*	KL220	MH752066	KX090868	KX090817
* Thuemenidium atropurpureum*	ILLS 61044	JQ256427	–	JQ256441
Mniaciaciae				
* Mniaecia albida*	CBS 126302	MH863969	–	MH875424
	CBS 126301	MH863968	–	MH875423
	M193	EU940204	EU940055	EU940128
* Mniaecia jungermanniae*	M145	EU940185	EU940036	EU940109
* Mniaecia nivea*	M167	EU940188	EU940042	EU940115
Tympanidiaceae				
* Aotearoamyces nothofagi*	PDD 95741	NR_164216	–	–
	PDD 106298	MG807392	MG807389	MG807388
	ICMP 21868	–	MG807390	MG807386
* Claussenomyces kirschsteinianus*	GMC2015-05-022	KY689631	KY689631	KY689631
	GM2014-11-122	KY689629	KY689629	KY689629
	GMC 2014-11-084	KY689628	KY689628	KY689628
	ICMP 21869	–	MG807391	MG807387
* Claussenomyces olivaceus*	NB-479	KY633590	–	KY633629
* Claussenomyces prasinulus*	CBS 111551	MN082653	–	MN082657
* Collopphorina africana*	CBS 120872	NR_119748	GQ154630	MK314588
	CBS:120879	GQ154571	GQ154631	GQ154610
* Tympanis confusa*	CBS 354.55	MK314568	–	–
* Tympanis tsugae*	CBS 369.55	MH857515	–	MH869054
**Phacidiales**				
Helicogoniaceae				
* Eleutheromyces subulatus*	CBS 458.88	NR_145309	EU754063	EU754162
	CBS 113.86	KJ710468	EU754062	KJ710444
* Gelatinipulvinella astraeicola*	NBRC 112540 Type	LC425040	LC434573	LC429381
* Geltingia associata*	Perez-Ortega 1039	KJ559540	KJ559584	KJ559562
* Gelatinopsis fungicola*	NBRC 112558	LC425051	LC434551	LC429387
Phacidiaceae				
* Allantophomopsis lunata*	CBS 137781	KR873229	–	KR873263
* Bacilliformis hyalinus*	MFLU 18-1811	MK584997	–	MK591951
* Bulgaria inquinans*	AFTOL_ID_916	KJ663831	DQ471008	DQ470960
* Darkera picea*	CPC 23897	NR_132906	KM108446	KM108397
* Phacidium lacerum*	CBS 130.30	KJ663841	–	KJ663882
* Phacidium lauri*	CBS 308.68	KJ663850	–	KJ663891
* Potebniamyces pyri*	AFTOL-ID 744	DQ491510	DQ470997	DQ470949
**Thelebolales**				
Holwayaceae				
* Holwaya mucida*	–	DQ257357	DQ257355	DQ257356
	CNF 2/8749	OM282975		OM282978
	CBS:630.85	MN082656		MN082660
* Crinula caliciiformis*	AFTOL_ID 272	KT225524	AY544729	AY544680
Unresolved taxa				
	**MUT 6736**	**OQ911372**	**OR145145**	**OQ920106**
	**MUT 6737**	**OQ911371**	**OR145144**	**OQ920105**
	**MUT 6739**	**OQ911369**	**OR145143**	**OQ920103**
**Gleoglossales**				
Gleoglossaceae				
* Sarcoleotia globosa*	OSC6363	AY789410	–	AY789409
	HMAS71956	AY789300	AY789298	AY789299

Newly generated sequences relative to the putative new taxa are in bold.

**Figure 5:**
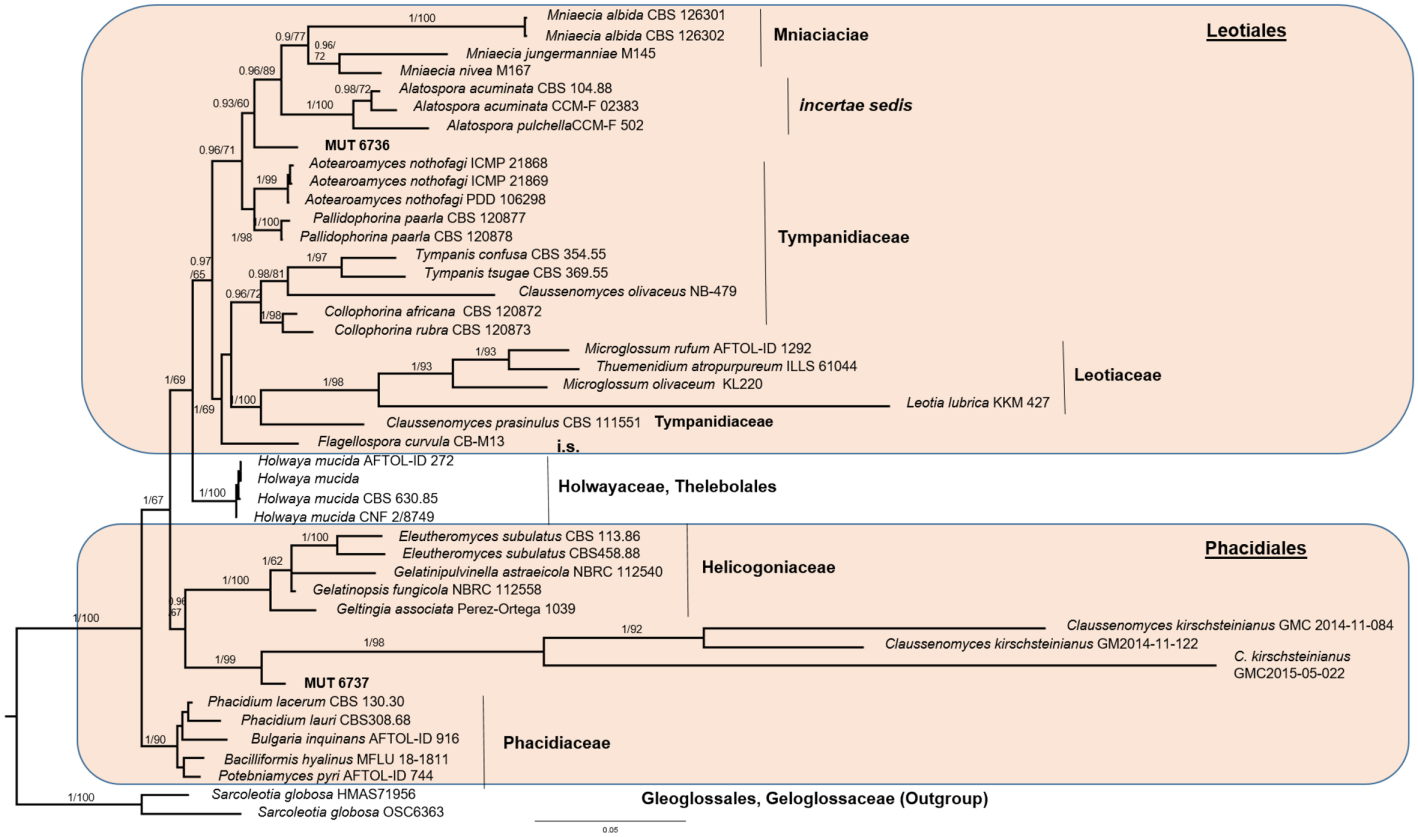
Bayesian phylogram of target families of Leotiales and Phacidiales (Leotiomycetes) based on a combined nrITS, and nrLSU dataset. The tree is rooted to Gleoglossales. Branch numbers indicate BYPP/BS values; Bar = expected changes per site (0.05).

For Leotiales/Phacidiales, the three-loci dataset consisted of 44 taxa, including MUT 6736 and MUT 6737, that represented 24 genera and 32 species (Table 3). The dataset, combining nrITS, nrSSU and nrLSU, had an aligned length of 2,193 characters, of which 1,126 were conserved, 516 were parsimony uninformative and 551 were parsimony informative and (TL = 1055, CI = 0.429298, RI = 0.699357, HI = 0.570702). The strains under investigation, namely MUT 6736 and 6737 fell respectively into the orders Leotiales (BYPP = 0.97, BS = 60%) and Phacidiales (BYPP = 0.96, BS = 65%), and likewise MUT 6739, potentially represented new lineages (Fig. 5).

## Discussion

In accordance with other studies that focused on the mycobiota of subterranean environments, Ascomycota was the most abundant phylum^26,32^. Dothideomycetes, Eurotiomycetes and Sordariomycetes dominated the sediments, which is consistent with the culture-dependent and culture-independent approaches that have been employed to investigate cave mycobiota worldwide^31,35^. The same classes prevailed in eight wild caves of the Great Basin National Park, in Nevada^36^. Surprisingly, these results were in contrast with the findings of Biagioli et al.^20^, who described a dominance of Sordariomycetes and Saccharomycetes in Bossea and of Sordariomycetes and Mortierellomycetes in Costacalda. In our study, a small percentage of Saccharomycetes was observed in S2, while Mortierellomycetes were not detected, which can only be explained by considering the different methodology applied. Usually, culturomics (i.e. the isolation of microorganisms in axenic cultures) overestimates highly sporulating fungi, going to the detriment of yeasts or poorly sporulating micromycetes. In fact, the broad diffusion of the genera *Aspergillus* and *Penicillium* (Aspergillaceae, Eurotiales) and of *Cladosporium* (Cladosporiaceae, Cladosporiales) is most probably due to their high adaptability and diffusion ability. In addition, contrary to *Penicillium*, species of *Aspergillus* are not found neither in S4 nor in CC, thus establishing the dependency of fungal distribution on the biotope characteristics. For instance, an increased humidity is not suitable for the genus *Aspergillus*^32^.

The sediments of Bossea cave displayed a higher number of taxa in respect to Costacalda (224 isolates—60 taxa vs 26 isolates—14 taxa). However, biodiversity indices were higher in Costacalda due the lower number of taxa and individuals found therein. Indeed, the mycobiomes described through the distribution of unique Amplicon Sequence Variants (ASVs) were more complex in Bossea and in three other show-caves than in the natural wild cave of Costacalda^20^. This difference is likely due to the flow of visitors occurring in the show-cave: while Costacalda is an undisturbed natural cave, Bossea cave receives almost 16,000 tourists per year^21^ who possibly serve as a vehicle of fungal propagules and organic material^26,37^. Moreover, considering the temperature, we can hypothesise that only psychrophile/psychrotolerant fungi detected exclusively at 10 °C are cave dwellers. Interestingly, the putative new taxa were isolated at 10 °C and did not grow at 25 °C. This underlines once again the importance of mimicking the natural environment in terms of temperature (10 °C) and/or oligotrophy (SNA medium), in order to optimise the recovery of the autochthonous mycoflora^38^. Had we applied standard methods (e.g. PDA at 25 °C), these taxa would have never been discovered. A broad investigation from Zang et al.^25^ supports this idea: by using only one incubation temperature (25 °C) and one culture medium (1/4 PDA) to analyse rocks, sediments and water from 13 karstic caves in China, the authors described a number of novel taxa more than twofold less than ours.

In line with other studies that assume a unique mycobiota for each individual cave^26,32^, only a few species were shared between Bossea and Costacalda, namely *A. pullulans*, *C. allicinum*, *C. cladosporioides*, *C. pseudocladosoprioides* and *P. pannorum*. Of these, two were exclusively isolated at 10 °C, while the others appreciated both 10 °C and 25 °C, thus indicating that they may represent an endemic component of the subterranean environment. Remarkable is also the high percentage of taxa (35%) reported for the first time from hypogean habitats worldwide (Table 2).

Despite having only five taxa in common (Fig. 2B), the fungal communities of the four sectors of Bossea were not significantly different among each other. One of the reasons for this lies in the unevenness of the samples that can be visualised in the PCO (Fig. 3). Furthermore, the classical division of caves in entrance, twilight and dark zone is not applicable here, since, with the exception of S4 that is truly a dark zone close to tourists, S1, S2 and S3, are all artificially illuminated and sediments have been on average collected from equal distances from the light sources. The distribution of micromycetes in caves is in fact influenced by biotic and abiotic factors^39^ that are here silenced by the constant flow of visitors and by the presence of artificial lights. On the contrary, a significant variation in species composition is observed between the mycobiota of Bossea and Costacalda, two caves located only a few kilometres apart. Beside the absence of visitors and the lack of artificial lamps, the narrow opening of Costacalda cave (personal communication by Dr Piano) may not be an easy entrance for animals and organic materials through currents, thus decreasing the transport and settling of “outsider propagules”.

In an attempt to compare the culture-dependent with a culture-independent approach that targeted the ITS1 region, we ran a standalone blastn analysis (https://ftp.ncbi.nlm.nih.gov/blast/executables/blast+/LATEST/) keeping the newly generated nrITS sequences as a query and setting the fungal ASVs obtained from the caves of Bossea and Costacalda as local database^20^. Thirty-five out of 69 taxa—including MUT 6736, MUT 6737 and MUT 6739—were found with both methods (Table 4). The analysis of environmental DNA by metabarcoding, could also lead to the amplification of dead organisms, that is why several taxa identified by ITS and found in one or two sectors, had correspondent ASVs in more sampling sites. Clearly, this analysis could be run only with those taxa whose molecular identification relied on ITS sequences. In addition, a few lineages such as Blastocladiomycota, Chytridiomycota, Rozellomycota or Mucoromycota were observed only through a culture independent-method. This testify how the two different methodologies are complementary and not comparable: while culturomics allows a deepest investigation, with the availability of the live organisms, a wider perspective can be achieved through metabarcoding, giving the possibility to improve techniques aimed at increasing the probability to isolate taxonomic groups that require specific conditions to grow.

**Table 4:** Matching of fungal taxa detected by culture-dependent approach with fungal ASVs from culture-independent approach (Similarity: 90–100%).✓

Culturomics						Metabarcoding						Sim %
Taxon	S1	S2	S3	S4	CC	Fungal taxa (ASV)	S1	S2	S3	S4	CC	
*Alternaria alternata*				✕		*Alternaria* (ASV249, ASV2430, ASV1466, ASV5203, ASV6914)	✕	✕	✕	✕	✕	99–100
*Anopodium ampullaceum*	✕					Lasiosphaeriaceae (ASV313, ASV11, ASV1476)	✕	✕	✕	✕	✕	98–100
*Apiotrichum dulcitum*		✕				*Apiotrichum* (ASV6, ASV4577, ASV2372, ASV268, ASV2982)	✕	✕	✕	✕	✕	98–100
*Arthroderma terrestre*	✕					Arthrodermataceae, *Trichophyton* (ASV2632, ASV7547, ASV4457 )	✕		✕	✕		98–100
*Arthroderma uncinatum*		✕				*Arthroderma* (ASV2378)	✕		✕		✕	100
*Aureobasidium pullulans*	✕				✕	Aureobasidiaceae, *Aureobasidium* (ASV575, ASV4267, ASV7844, ASV7358 )		✕	✕		✕	99–100
*Botrytis cinerea*			✕			*Botryotinia* (ASV7216)				✕		98
*Cadophora de✕trinospora*			✕			*Cadophora* (ASV1800)	✕		✕	✕		100
*Cephalotrichum domesticum*			✕			*Cephalotrichum* (ASV734, ASV634, ASV170, ASV23)	✕	✕	✕	✕	✕	98–100
*Cephalotrichum longicollum*				✕		*Cephalotrichum* (ASV634, ASV734, ASV23	✕	✕	✕	✕	✕	98–99
*Cladosporium allicinum*					✕	*Cladosporium* (ASV5598, ASV296, ASV3335)	✕	✕	✕	✕	✕	99
*Cladosporium cladosporioides*	✕					Ascomycota, *Cladosporium* (ASV41, ASV398, ASV3661, ASV353, ASV3343, ASV215, ASV415, ASV516, ASV1353	✕	✕	✕	✕	✕	98–100
*Cordyceps farinosa*					✕	*Isaria* (ASV2450)	*✕*				✕	100
*Epicoccum nigrum*	✕	✕	✕			*Epicoccum* (ASV173, ASV6622, ASV2726)	✕	✕	✕	✕	✕	99–100
*Furcasterigmium furcatum*			✕			*Acremonium, Gibellulopsis, Cephalosporium* (ASV455, ASV614, ASV1953, ASV2745, ASV1296)	✕	✕	✕	✕	✕	98–100
*Fusarium* sp.				✕		*Gibberella* (ASV4526, ASV158, ASV5204, ASV4897, ASV3298, ASV2105)	✕	✕	✕	✕	✕	98–100
** Leotiales sp**.			**✕**			**Leotiomycetes (ASV434, ASV3854)**		**✕**	**✕**	**✕**		**99–100**
*Lasionectria hilhorstii*			✕			Nectriaceae, Ascomycota (ASV199, ASV1877)	✕		✕	✕	✕	99–100
*Lecanicillium coprophilum*		✕				*Lecanicillium* (ASV4613, ASV6092)	✕		✕	✕		99–100
** Phacidiales sp.**				**✕**		**Helotiales (ASV166, ASV96)**	✕	✕	✕	✕		100
*Mammaria echinobotryoides*	✕		✕			*Cercophora* (ASV58, ASV1424, ASV219, ASV627)	✕	✕	✕	✕	✕	98–100
** Neobulgaria sp.**				✕		Helotiales, *Neobulgaria* (ASV208, ASV566, ASV1829)	✕	✕	✕	✕		98–100
*Oidiodendron tenuissimum*			✕			*Oidiodendron* (ASV558)	✕		✕	✕		100
*Paracremonium variiforme*		✕		✕		Nectriaceae (ASV65, ASV77, ASV1487)	✕	✕	✕	✕	✕	98–100
*Penicillium expansum*			✕			*Penicillium* (ASV177)	✕	✕	✕	✕	✕	100
*Penicillium glandicola*					✕	*Penicillium* (ASV143, ASV529, ASV196, ASV257, ASV338, ASV1126, ASV572, ASV2411 )	*✕*	*✕*	*✕*	*✕*	✕	98–100
*Psathyrella candolleana*		✕				*Psathyrella* (ASV3288, ASV2917)	✕					99–100
*Pseudogymnoascus pannorum*	✕	✕	✕		✕	*Pseudogymnoascus* (ASV118, ASV103, ASV828, ASV601, ASV350, ASV590, ASV167, ASV1100	✕	✕	✕	✕	✕	98–100
*Sporothrix inflata*	✕					*Sporothrix* (ASV371, ASV4050)	✕			✕		98–99
*Stephanonectria keithii*			✕			*Stephanonectria* ( ASV1118, ASV3677)	✕		✕			99
*Tetracladium globosum*				✕		*Tetracladium* (ASV691, ASV5298, ASV36, ASV4432, ASV130, ASV597, ASV264, ASV508, ASV624)	✕	✕	✕	✕	✕	98–100
*Tolypocladium cylindrosporum*	✕				✕	*Tolypocladium* (ASV2777, ASV69, ASV7850, ASV7057, ASV5653, ASV4447, ASV417, ASV3074, ASV1751, ASV1477, ASV1157, ASV6918, ASV6203, ASV5227, ASV2494, ASV4750, ASV5086 )	✕	✕	✕	✕	98–100	
*Trichocladium asperum*			✕			Trichocladium (ASV17, ASV165)	✕	✕	✕	✕	✕	98–99
*Trichosporiella cerebriformis*			✕			*Tetracladium* (ASV36, ASV691, ASV4432, ASV5298, ASV130, ASV597, ASV264, ASV508, ASV624, ASV180, ASV616)	✕	✕	✕	✕	✕	98–99
*Volutella ciliata*		✕				*Volutella* (ASV100, ASV791, ASV361, ASV413)	✕		✕	✕	✕	98–100

Newly generated sequences relative to the putative new taxa are in bold.

As for the unresolved taxa MUT 6736, MUT6737 and MUT 6739, some careful consideration must be drawn. While the phylogenetic inference of *Neobulgaria* sp. was clear, the placement of MUT 6736 and MUT 6737 was more complex and obscure. With the aid of the three ribosomal markers MUT 6736 resulted affiliated to *incertae sedis* located between Mniaciaciae and Tympanidiaceae of the order Leotiales while MUT 6737 clustered with three strains of *Claussenomyces kirschsteinianus*. *Claussenomyces* is a polyphyletic genus of the family Tympanidiaceae^40,41^ that is dispersed between the orders Leotiales and Phacidiales^42^. In this last case, the clear distance from *C. kirschsteinianus*, the paucity of available sequences in public databases (only the reported three strains are accessible), together with the absence of a monograph of the genus and the description of the species, represented an obstacle. We also need to consider that several species morphologically described, have never been molecularly typified. On the other hand, despite using different culture media, temperatures and long incubation periods (up to 4 months), the three strains remained sterile, making a morphological description complicated. For all these reasons, the identification of MUT 6739 (*Neobulgaria* sp.), MUT 6736 (*Leotiales* sp.) and MUT 6737 (*Phacidiales* sp.) did not go beyond genus and order level, respectively. A deeper investigation would be sought to solve this matter. Indeed, additional isolations are necessary to recover strains sitting in the same phylogenetic position before proceeding to the formal description of new taxa.

As mentioned above, noteworthy is the detection of the three putative novel taxa through the culture-independent approach, indicating the diffusion of these organisms in the subterranean environment and their adaptability to specific conditions.

## Conclusion

With this work, we detailed and compared the cultivable fungal diversity inhabiting two karstic caves, wild vs touristic, in Italy. Human fluxes seem to deeply influence the mycobiota composition of the show-cave, being richer in terms of fungal species diversity. Regardless of whether they are touristic or wild caves, the hypogean habitats are here confirmed as a reservoir of still undescribed fungi (Zhang et al. 2017; Zhang et al. 2021). Indeed, potentially novel lineages were detected in our investigation and a high percentage of taxa was observed for the first time in subterranean environments. Since fungi from extreme environments can represent a resource of biotechnological importance, offering several benefits (i.e. production of novel bioactive metabolites, mycoremediation, etc.), exploring and reporting the fungal biodiversity from caves becomes more and more urgent.

## Materials and methods

### Sampling

Samples of sediments were harvested from two caves that are part of an extensive karst system located in the Maritime Alps complex (Piedmont region, Italy), namely Bossea (44°14′31.0′′N; 7°50′24.0′′E) and Costacalda (44°14′24.8′′N; 7°50′54.9′′E) caves.

Sediments were collected in summer 2020 at increasing distances from the cave entrance and, in order to represent a gradient of anthropic pressure (High, Medium and Low pressure), at three increasing distances from the touristic path, as detailly described by Piano et al.^21^. Briefly, for Bossea, four sectors (1–4) were identified: Sector 1 was close to the cave entrance and, likewise Sectors 2 and 3, was open to the public, contrary to the deepest Sector 4, (indicated as S1, S2, S3 and S4, respectively). S4 is the undisturbed part of the cave, being closed to visitors and only sporadically frequented by speleologists. For the natural cave of Costacalda (CC), three sites were sampled in the accessible area only and treated as a whole to gain the best detection possible of mycodiversity of this portion of the cave.

For each sampling point, 3 replicates, up to 5 cm depth, were collected using sterile Falcon tubes (50 mL). Samples were stored in a cooler-bag until arrival at the laboratory, where the 3 replicates were pooled and homogenized. Overall, the number of samples analysed was 12 for Bossea and 3 for Costacalda.

Preliminary analyses performed on Bossea cave, revealed that along increasing distance from the entrance (i.e. within each Sector) no significant difference occurred among the three sampling sites (High, Medium and Low pressure). Therefore, only Sectors as a whole were considered.

### Fungal isolation

In order to remove coarse rock debris, sediments were sieved. Following, each sample was serially diluted in sterile 0.9% NaCl; 1 mL of the 1:50,000 dilution was placed onto Petri dishes (15 cm Ø) containing Potato Dextrose Agar (PDA; 39 g PDA- Sigma-Aldrich Saint Louis, USA—1 L H_2_O_d_). Furthermore, in an attempt to reproduce the natural conditions and to maximize the selection of endemic fungi, a minimal medium, namely Synthetic Nutrient-poor Agar (SNA: 1 g L^−1^ KH_2_PO_4_, 1 g L^−1^ KNO_3_, 0.5 g L^−1^ MgSO_4_ × 7H_2_O, 0.5 g L^−1^ KCl, 0.2 g L^−1^ glucose, 0.2 g L^−1^ saccharose, 18 g L^−1^ agar), was employed. Both media were supplemented with antibiotics (Gentamicin 80 mg L^−1^ and Tazobactam 100 mg L^−1^) to prevent bacterial growth. Plates were incubated at three different temperatures: 10 °C (representative of the caves)—to isolate psycrotholerant and/or psycrophilic fungi; 24 °C—to isolate mesophilics; and 37 °C—to isolate thermophiles and/or human opportunists. Five replicates per each condition were prepared. Colony forming units per gram of dry weight (CFU g^−1^dw) were recorded; strains were isolated in axenic culture and preserved on Malt Extract Agar slant at the *Mycotheca Universitatis Taurinensis.*

### Fungal identification

The strains isolated were identified by the mean of a polyphasic approach that combines morpho-physiological and molecular analysis, as follows. Fungi were first identified on the basis of macro- and microscopic features following specific taxonomical keys^43^. Next, molecular analyses were performed by amplifying and sequencing specific markers.

### DNA, PCR amplification and data assembling

Fresh mycelium was gently scraped from Malt Extract Agar (MEA: 20 g L^−1^ malt extract, 20 g L^−1^ glucose, 2 g L^−1^ peptone, 20 g L^−1^ agar) plates, transferred to a 2 mL Eppendorf tube and disrupted by the mean of a MM400 tissue lyzer (Retsch GmbH, Haan, Germany). The manufacturer’s instruction of a NucleoSpin Kit (Macherey Nagel GmbH, Duren, DE, USA) were followed to extract genomic DNA. The quality and quantity of DNA were measured spectrophotometrically (Infinite 200 PRO NanoQuant; Tecan, Männedorf, Switzerland); samples were then stores at − 20 °C.

The partial sequences of specific markers were amplified in a T100 Thermal Cycler (Bio-Rad, Hercules, CA, USA). The internal transcribed spacer, including the 5.8S rDNA gene (nrITS), the 28S large ribosomal subunit (nrLSU) and the 18S small ribosomal subunit (nrSSU), were amplified using primer pairsITS1/ITS4^44^, LR0R/LR7^45^, and NS1/NS4^44^, respectively. NL1/NL4 were used to amplify the D1/D2 region of LSU^46^ in yeasts. The β-tubulin (β-tub; for the genera *Aspergillus* and *Penicliium*) and the α-actin (α-act; for the genus *Cladosporium*) genes were amplified using respectively primer pairs Bt2a/Bt2b^47^ and ACT512F/ACT783R^48^, while fRPB2-5F/fPB2-7R^49^ served to amplify the largest and second-largest subunits of RNA polymerase II (RPB2). Reactions occurred in 50 µL final volume and consisted of 20–40 ng DNA template, 10 × PCR Buffer (15 mM MgCl_2_, 500 mM KCl, 100 mMTris-HCl, pH 8.3), 200 µM each dNTP, 1 µM each primer, and 2.5 U Taq DNA Polymerase (Qiagen, Chatsworth, CA, USA). Negative controls with no DNA template were included. Amplicons, together with a GelPilot 1 kb plus DNA Ladder, were visualized on a1.5% agarose gel stained with SYBR™ Safe (Thermo Fisher Scientific, USA); PCR products were purified and sequenced at the Macrogen Europe Laboratory (Madrid, Spain). The resulting Applied Biosystem (ABI) chromatograms were inspected, trimmed, and assembled to obtain consensus sequences using Sequencer 5.2 (GeneCodes Corporation, Ann Arbor, MI, USA, http://www.genecodes.com). Newly generated sequences were compared to those available in public databases (GenBank—nblast; mismatch 1/-2; gap costs linear; Mycobank) and deposited at NCBI.

Sterile mycelia and strains with morphological features that did not match any available species description and showed low sequence similarity with those available in public databases were further characterised through phylogenetic inference.

### Phylogenetic analysis

Two dataset consisting of nrITS and nrLSU Gelatinodiscaceae and in nrSSU, nrITS and nrLSU for Leotiales/Phacidiales, were assembled on the basis of BLASTn results and of the available phylogenetic studies focused on Leotiomycetes^40,41,50,51^. Reference sequences were obtained from GenBank. Sequences were aligned using MUSCLE (default conditions for gap openings and gap extension penalties), implemented in MEGA 7 (Molecular Evolutionary Genetics Analysis), visually inspected, and manually trimmed to delimit and discard ambiguously aligned regions. Individual alignments were concatenated into a single data matrix with Sequence-Matrix^52^ since no incongruence was observed among single-loci phylogenetic trees. The best evolutionary model under the Akaike Information Criterion (AIC) was determined with jModelTest 2^53^. Phylogenetic inference was estimated using Maximum Likehood(ML) and Bayesian Inference (BI) criteria. The ML analysis was generated using RAxML v.8.1.2^54^ under under GTR + I + G evolutionary model and 1000 bootstrap replicates. Support values from bootstrapping runs (BS) were mapped on the global best tree using the “-f a” option of RAxML and “- × 12,345” as a random seed to invoke the novel rapid bootstrapping algorithm. BI was performed with MrBayes 3.2.2^55^ with the same substitution model. The alignment was run for 10 million generations with two independent runs each, containing four Markov Chains Monte Carlo (MCMC) and sampling every 100 iterations. The first 25% of generated trees were discarded as “burn-in”. A consensus tree was generated using the “sumt” function of MrBayes and Bayesian posterior probabilities (BYPP) were calculated. Consensus trees were visualized in FigTree v. 1.4.2 (http://tree.bio.ed.ac.uk/software/figtree). *Sarcoleotia globosa* and *Chlorociboria aeruginosa* served as outgroups for the two trees. Due to a topological similarity of the two resulting trees, only Bayesian analysis with BS and BYPP values was reported.

### Morphological analysis

The strains MUT 6736, MUT 6737, MUT 6739 were pre-grow on MEA for one month at 10 °C prior to inoculation in triplicate onto new Petri dishes (9 cm Ø) containing (i) MEA, (ii) Oatmeal Agar (OA; 30 g L^−1^ oatmeal, 20 g agar in 1 L of sea water), or iii) PDA. In an attempt to induce sporulation, Petri dishes were incubated at 5, 10, 15 and 24 °C up to four months. The colony growth was monitored periodically for 28 days.

### Statistical analysis

Significant differences among mycobiota were evaluated by applying the PERmutational Multivariate ANalysis Of Variance (PERMANOVA; pseudo-F index; *p* < 0.05) and visualised by the Principal Coordinate Analysis (PCO). The contribution of single species (in percentage) to the diversity observed within and between groups was assessed by SIMilarity PERcentage (SIMPER) analysis. The biodiversity within sampling sites and matrices was evaluated by calculating the Shannon–Weaver index (H′), the Simpson index (1-Lambda), and the Pielou’s evenness (J′). The analyses were performed with the statistical package PRIMER 7 (Plymouth Routines in Multivariate Ecological Research, Albany Auckland, New Zealand).

### Supplementary Information

Supplementary Information.

## Data Availability

All newly generated nucleotide sequences presented in this work have been deposited in GenBank (https://www.ncbi.nlm.nih.gov/). Accession numbers are listed in the Supplementary materials. All data analysed during this study are included in the article.
