# Supplementary material for: Cultivable fungal diversity in two karstic caves in Italy: under-investigated habitats as source of putative novel taxa
Source: Sci Rep. 2024 Feb 20;14:4164. doi: 10.1038/s41598-024-54548-1 (PMC10879487; doi:10.1038/s41598-024-54548-1)
Supplement: Supplementary file 1 — Supplementary Information. [file 41598_2024_54548_MOESM1_ESM.pdf]

Accession Numbers of the newly generated sequences

ITS:OQ911379, OQ911387, OQ911386, OQ911388, OQ911381, OQ911389, OQ911390, OQ954843, OQ911374, OQ911370, OR085842, OQ911391, OQ954847, OQ911375, OQ911382, OQ911371, OR085841,OR178928, OQ911372, OQ911392, OQ911393, OQ954842, OQ911369, OQ911394, OR178929, OQ911383, OQ911380, OQ911395, OQ911384, OQ911396, OQ911385, OQ954844, OQ911397, OQ954845, OQ954846, OQ911376, OQ911377, OQ911398, OQ911399, OQ911373, OQ911378, OQ911400
LSU: OQ920104, OQ920105, OQ920106, OQ920103, OQ920107; SSU: OR145144, OR145145, OR145143
ACT: OR189139, OR189140, OR189138, OR189137, OR189141, OR189142, OR189143, OR189144
β-TUB: OR217446, OR217444, OR217447, OR217448, OR217445, OR217449, OR334359, OR217442, OR217443

Table S1. Percentage of similarity derived by Blastn analyses of the four genetic markers relative to the three unresolved strains.

	**MUT 6736**	**MUT 6737**	**MUT 6739**
**nrITS**	*Aotearomyces nothofagi* 93%	Fungal sp. 96%	*Neobulgaria* sp. 99%
**nrSSU**	*Leotiomycetes* sp. 99%	*Leotiomycetes* sp. 98%	*Neobulgaria* sp. 99
**nrLSU**	*Pallidoporina paarla* 99%	*Claussenomyces* sp. 92%	*Helotiales* sp. 83%
**RPB2**	*Chlorociboria aeruginosa* 76%	*Helotiales* sp. 98%	*Neobulgaria koningiana* 97%
